# DNA methylation patterns of circadian and ultradian genes are altered in the peripheral blood of patients with hidradenitis suppurativa

**DOI:** 10.3389/fimmu.2024.1475424

**Published:** 2024-11-26

**Authors:** Uppala Radhakrishna, Uppala Ratnamala, Devendrasinh D. Jhala, Lavanya V. Uppala, Aaren Vedangi, Nazia Saiyed, Sushma R. Shah, Maulikkumar Patel, Rakesh M. Rawal, Tommaso Mazza, Gregor B. E. Jemec, Gianluigi Mazzoccoli, Giovanni Damiani

**Affiliations:** ^1^ Department of Anesthesiology and Perioperative Medicine, University of Pittsburgh, Pittsburgh, PA, United States; ^2^ Department of Life Sciences, School of Sciences, Gujarat University, Ahmedabad, India; ^3^ Department of Zoology, School of Sciences, Gujarat University, Ahmedabad, India; ^4^ College of Information Science and Technology, Peter Kiewit Institute, The University of Nebraska at Omaha, Omaha, NE, United States; ^5^ Department of Clinical Research, KIMS ICON Hospital, A Unit of ICON Krishi Institute Medical Sciences, Visakhapatnam, India; ^6^ Department of Obstetrics and Gynecology, Beaumont Health, Royal Oak, MI, United States; ^7^ Department of Obstetrics and Gynecology, BJ Medical College Institute of Medical Post-Graduate Studies and Research, Ahmedabad, India; ^8^ Bioinformatics Unit, IRCCS “Casa Sollievo della Sofferenza”, Opera di Padre Pio da Pietrelcina, San Giovanni Rotondo, FG, Italy; ^9^ Department of Dermatology, Zealand University Hospital, Roskilde, Denmark; ^10^ Department of Medical Sciences, Division of Internal Medicine and Chronobiology Laboratory, Fondazione IRCCS “Casa Sollievo della Sofferenza”, San Giovanni Rotondo, FG, Italy; ^11^ Department of Biomedical, Surgical and Dental Sciences, University of Milan, Milan, Italy; ^12^ Italian Center of Precision Medicine and Chronic Inflammation, University of Milan, Milan, Italy

**Keywords:** epigenetics, hidradenitis suppurativa, biological clock, circadian, ultradian, rhythmicity, chronic skin disorders

## Abstract

**Background:**

Hidradenitis suppurativa (HS) is a chronic inflammatory skin condition that affects hair follicles in areas with apocrine sweat glands, such as the underarms, groin, and buttocks. The pathogenesis of HS is not fully understood, but considering the key role played by the biological clock in the control of immune/inflammatory processes the derangement of circadian and ultradian pathways could be hypothesized.

**Methods:**

We analyzed genome-wide DNA methylation patterns in peripheral blood from 24 HS cases and 24 controls using the Infinium HumanMethylation450 BeadChip array (Illumina), followed by bioinformatics and statistical analyses.

**Results:**

We found that several circadian and ultradian genes were differentially methylated in HS patients, predominantly exhibiting hypomethylation. These genes were enriched in pathways such as MAPK and WNT cascades, acute phase response, cytokine release, inflammation, innate immune response, xenobiotic detoxification, and oxidative stress response.

**Conclusion:**

Altered DNA methylation patterns of genes related to circadian and ultradian pathways could contribute to immune system derangement and inflammatory processes chronicization in addition to other comorbidities hallmarking HS onset and progression, at the same time representing possible druggable targets.

## Introduction

Hidradenitis suppurativa (HS) is a chronic inflammatory dermatosis with unclear pathogenesis that severely impacts patients’ quality of life (1, 2). Due to this limited understanding, treatment is not well-defined and is shifting from broad-spectrum antibiotics to targeting TNF-alpha and IL-17/23 pathways (1). Similar to the pathogenesis of psoriasis, which involves dysregulated immune cell function and uncontrolled keratinocyte proliferation/differentiation, the altered balance between T-reg and Th1/Th17 cells is considered crucial in HS development and inflammation maintenance. Recently, B-cells also have been identified as a potential therapeutic target (3–5). Genetics plays a key role in HS pathogenesis, primarily through mutations in gamma-secretase complex genes like NCSTN, PSENEN, and PSEN1 (6, 7). While these mutations are common in familial cases, some are also seen in sporadic cases. Furthermore, various studies have identified multiple signaling pathways related to inflammation, epithelial differentiation, and metabolic dysregulation in HS (8). The genetic heterogeneity of HS underscores its complex inheritance patterns, contributing to variability in clinical presentation and treatment response, and highlighting the need for personalized therapeutic approaches (9, 10). The epigenetic landscape of HS is influenced by variations in microRNAs, cytokines, cytochromes, long non-coding RNAs (lncRNAs) (11–14), and other factors. These variations affect gene regulation by altering nucleosome structure and stability, which in turn impacts DNA methylation patterns (15).

All sections and components of the immune system are genetically and epigenetically regulated by the biological clock (16–18). Its disruption contributes to HS-related comorbidities like diabetes, obesity, metabolic syndromes, and sleep disorders (19), hypertension, addiction (20), depression (21), chronic pain (22, 23), body mass index (24), thyroid dysfunction (25, 26), traumatic stress, early aging (27), cognitive impairments, cancer, hormone secretion, cardiovascular health, glucose homeostasis, neurological disorders, body temperature, and immune response and inflammation (28).

The relationship between the biological clock and epigenetic control of gene transcription is an interesting area of study that may provide insights into the underlying molecular mechanisms of different diseases (chronomedicine). Accordingly, understanding the role of biological clock-related epigenetic modifications may allow to development of new therapeutic approaches that could maximize responsiveness while minimizing side effects (chronotherapy) (29). Circadian and ultradian genes encode functional and structural proteins that are critical for all cell processes and tissue functions. Genomic, genetic, and epigenetic changes of circadian and ultradian genes play crucial roles in metabolic, inflammatory, degenerative, and neoplastic diseases. Few studies have focused on the disruption of the circadian clock circuitry in pathological conditions from an epigenetic point of view (30), and no study addressed this issue in the setting of HS.

We aimed to identify epigenetically dysregulated transcripts that enrich circadian and ultradian pathways in HS patients and to explore their potential role in HS pathogenesis.

## Materials and methods

### Ethics statement

The study was approved by the Institutional Review Board of Beaumont Health System, Royal Oak, MI, USA (HIC code #: 2015-172, May 21^st^, 2015) and all the enrolled participants gave written informed consent. This study complies with the Declaration of Helsinki and was performed according to ethics committee approval.

### Hidradenitis suppurativa sample selection

Patients with HS had received diagnostic assessment at Gujarat University India using the visual-aided questionnaire for self-assessment of HS (31), and were subsequently examined by three independent board-certified dermatologists (RR, DGS, TM). The diagnosis was established following the Dessau criteria for HS (32) and samples were carefully assessed and described in line with European Hidradenitis Suppurative Foundation (EHSF) guidance (33). Disease severity was determined using the Hurley score (34), Hidradenitis Suppurativa Severity Score System (IHS4) (35), and Autoinflammatory disease damage index (ADDI) (19).

### Inclusion criteria

Adult patients (>20 years) with a diagnosis of HS with a duration greater than 5 years, b) at least a Hurley II severity, c) IHS4>3 points, d) ADDI<3 points, e) newly diagnosed (<3 months), f) untreated for 6 months.

### Exclusion criteria

a) syndromic HS, b) smoking, c) fasting regimens (36) and/or particular diet different from omnivore, and d) alcohol abuse (Alcohol Use Disorders Identification Test (AUDIT) >7 points), e) drug addiction, f) use of concomitant medications, including contraceptives, and foods cytokine inducers (i.e. grapefruits) g) treated for HS, h) chronic inflammatory/infectious diseases, history in the previous 5 years of cancer, m) persons unable to provide informed consent for any reason. No evident differences in age, smoking habits, addiction history, or body mass index (BMI) were present between the individuals with HS and those in the control group.

### DNA methylation analysis

DNA was extracted from the peripheral blood samples collected in the early morning from 24 HS patients and 24 matched controls for parental genetic screening to be performed at the Department of Obstetrics and Gynecology, VS hospital, India. The use of biological leftovers in the USA was carried out after obtaining approval from the local Ethics Committee (HIC code #: 2015-172, May 21st, 2015), according to the most recent ethical guidelines regarding the use of unused specimens in clinical laboratories. The details of specimen collection and preservation were previously published (11). From each sample 400 ng DNA was sodium bisulfite-treated using the EZ-96 DNA methylation kit (Zymo Research, CA, USA) according to the manufacturer’s standard protocol. Genome-wide methylation analysis was performed using the Infinium Human Methylation 450K BeadChip (Illumina, CA, USA) to detect methylation patterns. The Infinium Human Methylation 450K BeadChip enables the evaluation of the methylation status of over 450,000 CpG sites across the genome. The samples were randomized concerning sex, center, and disease status to avoid batch effects, and processed in a single batch BeadChips. A detailed description of the methodology has been previously reported (37, 38). Detailed statistical, bioinformatics, computational biology, Gene Ontology (GO), and KEGG pathway analyses were performed. The top differentially methylated genes were enriched for specific pathways and overlaid with genes identified in previous studies. We removed low-quality data with detection p-values > 0.01.

### Circadian genes prediction

We searched for predicted circadian transcripts using the Circadian Gene DataBase (CGDB, http://cgdb.biocuckoo.org), a database of circadian genes (39). Multiple predicted targets were identified and listed in the previously generated genome-wide methylation data.

### Circadian genes validation and ultradian genes prediction

To validate the results obtained using CGDB to predict circadian genes and to classify transcripts cycling at the second and third harmonic of circadian oscillation, bioinformatics analyses were performed using RhythmicDB (http://rhythmicdb.css-mendel.it) (40) a comprehensive database covering circadian and ultradian genes retrieved from several species, manually curated and analyzed by two different software packages, BioCycle and MetaCycle. In addition, we exploited a publicly available genomic dataset (GSE11923) obtained through high-temporal resolution profiling effected using Affymetrix arrays on liver specimens harvested every hour for 48 h from 6-week-old male C57BL/6J mice (Jackson), n = 3-5 per time point. The specimens were pooled and Fisher’s G-test at a false-discovery rate of < 0.05 and COSOPT were jointly exploited to recognize rhythmic transcripts, which were classified, according to the extent of the oscillation period, as circadian (24 ± 4 h) and ultradian (12 ± 2 h and 8 ± 1 h, respectively) (40). Array probe IDs/nucleotide sequences of 8-h, 12-h, and 24-h oscillating genes were listed by using BioDBnet (https://biodbnet-abcc.ncifcrf.gov/db/db2db.php).

### Heatmap

Heatmap was generated using the ComplexHeatmap module (v1.6.0) in the R package (v3.2.2) and considering individual sites of methylated CpG in the coding regions of 24-h cycling genes retrieved by CGDB. We applied Ward’s method for the *hierarchical cluster* analysis of samples (41). For comparison between HS and controls, CpG sites with FDR P-values ≤0.05 were considered significantly differentially methylated. Based on methylation levels at the most significantly differentially methylated CpG loci, the area under the receiver operating characteristic (AUC-ROC) was calculated.

### Principal component analysis

To improve interpretability and, at the same time, minimize the loss of information, Principal Component Analysis (PCA) was used to identify the key epigenetic differences between the two study groups outlined above. R function “prcomp” was used to compute principal components (PCs) used for the PCA distribution plot. The 3D PCA distribution plot was generated by using the R package “ggplot2”.

### Biological functional enrichment analysis

Gene Ontology (GO), and Kyoto Encyclopedia of Genes and Genomes (KEGG) pathway enrichment analyses were conducted with CpG methylation changes associated with HS with a statistical significance of p-value <0.05.

### STRING protein-protein interaction network

A Search Tool for Retrieval of Interacting Genes/Proteins (STRING) (http://string-db.org) was employed to seek potential interactions among differentially methylated circadian genes in HS patients. The STRING database contains known and predicted protein interactions that cover proteins from multiple organisms, either directly or indirectly through physical or indirect interactions. Genes associated with a differentially methylated CpG (FDR < 0.05) were entered into STRING and visualized in Cytoscape. To analyze the properties of the PPI network, only connected nodes were retained. The Biological Networks Gene Ontology tool was used to identify enriched gene ontology (GO) terms (42).

### Pathway analysis and functional annotation through REACTOME and IPA software

REACTOME pathway analysis of gene expression data was accomplished using various Bioconductor libraries, including Pathview, GOFunction, ReactomePA, and org.Hs.eg.db (42–44). Firstly, gene symbols were converted to Entrez IDs, removing unmapped components, before being filtered for enriched pathways, and mapped to a bar chart. REACTOME pathway analysis results allow for the specific comparison of up and downregulated components enriching the pathways, whereas the KEGG map shows how or where these components interact.

We also performed Ingenuity Pathway Analysis (IPA QIAGEN) to implement wide-ranging data analysis consenting to recognize investigational outcomes, to envisage downstream influences, and to recognize novel targets/biomarkers in the setting of HS. Disease, function, pathway, and functional network analyses were conducted using Ingenuity Pathway Analysis (IPA; QIAGEN, Redwood City, CA; www.qiagen.com/ingenuity). The in-silico functional enrichment analysis was conducted considering the entire Ingenuity Knowledge Base, Gene interactions were checked against it, and interacting genes were identified as network-eligible molecules, which were used, in turn, as seeds for generating networks. Networks were assigned scores, which were based on the number of network-eligible molecules they contained. Scores were based on the hypergeometric distribution and were calculated with the right-tailed Fisher’s exact test. The higher the score, the lower the probability of finding the observed number of network-eligible molecules in a network by chance. Moreover, the higher the interconnection among genes in a network, the higher the probability that the network represents significant biological functions. The completeness index (ρ) was computed to assess the extent of interconnectivity of each obtained network. Dense networks exhibited ρ ≥ 1, and sparse networks had ρ < 1. The more extreme the value of ρ, the denser or sparser the corresponding network.

### Statistical and bioinformatics analysis

Data (IDAT files) were normalized using Genome Studio software functional normalization and determined Cytosine methylation levels (ß-value) for each CpG site. Before analysis, we removed all CpG-probes that had missing ß-values. Differential methylation was assessed by comparing the ß-values for the cytosine at each CpG locus in HS versus controls. To avoid confounding factors, we removed probes associated with sex chromosomes, non-specific probes, and probes targeting CpG sites within 10 bp of SNPs (each of which listed dbSNP entries within 10 bp of the CpG site) (45–47). In addition, SNPs with a minor allele frequency ≤0.05 were only considered for forwarding analysis.

Significantly differentially methylated CpG sites between HS and controls were defined based on preset cutoff criteria FDR p<0.05. Multiple CpG sites within a gene were resolved by the selection of the CpG with the highest AUC ROC ranking and the lowest p-value. The p-value for methylation differences between case and control groups at each locus was calculated and Raw and FDR p-values corrected for multiple testing (Benjamini-Hochberg test) were calculated. AUC for combinations of loci was calculated using the ‘R’ program “ROCR” package (v3.5.0).

## Results

The demographic characteristics of the subjects enrolled in this study have been previously reported (48). As anticipated, there were no noticeable differences in age, smoking habits, addiction history, or body mass index (BMI) between the individuals with HS and those in the control group. **Supplementary Tables S1**–**S4** display the list of differentially methylated oscillating genes with the most significant FDR values across all criteria. In **Supplementary Figure S1**, we present the performance of four individual CpG loci based on their Area Under the Receiver Operating Curve (AUC-ROC) scores, showcasing their consistency for HS detection. In total, 66 CpG loci showed exceptional accuracy (AUC ≥0.90-1.00), and 189 showed good accuracy (AUC ≥0.80-0.89) for HS detection.

### Evaluation of heatmaps

**Figure 1:**
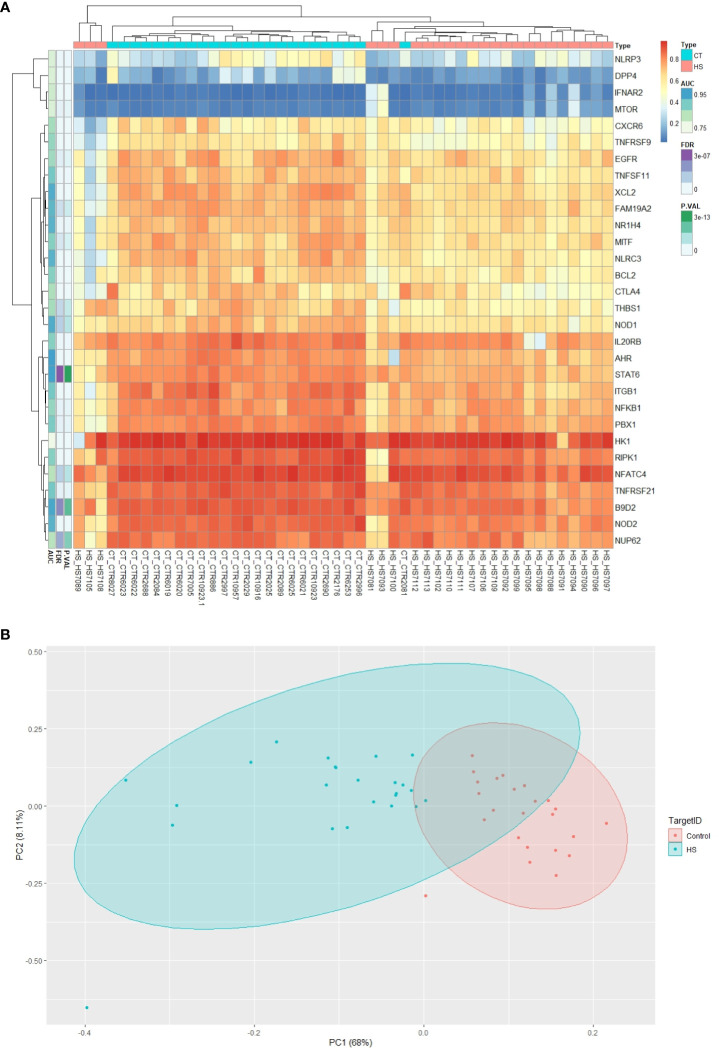
Graphical representation of differentially methylated circadian genes. **(A)** Comparative heatmap of differentially methylated CpG sites between HS and control groups. CpGs with low methylation levels appear green, while those with high methylation levels appear red. **(B)** Results of Principal Component Analysis (PCA) of circadian genes with CpG markers in HS and control groups.

Using CpG methylation markers related to circadian pathways, the heatmap clearly illustrates the existence of two distinct clusters of CpGs: one associated with HS patients and the other with the control group. This convincing data lends strong support to the idea that these methylation markers are remarkably reliable in distinguishing between HS patients and healthy subjects. In essence, our results validate the precision and effectiveness of these methylation markers in accurately discriminating between the two research groups (**Figure 1A**).

### Principal component analysis

PCA effectively facilitated clear differentiation between the patient groups with HS and the control group, providing strong evidence for the discriminative utility of the epigenetic markers displayed in **Figure 1B**.

### Circadian gene GO enrichment analysis

**Figure 2:**
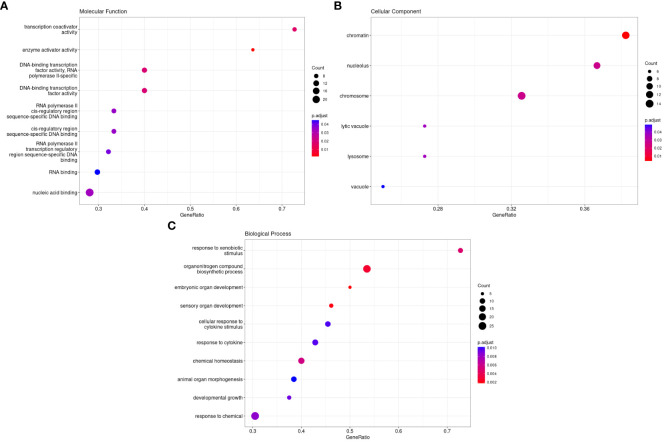
Gene ontology analysis of differently methylated genes in HS. **(A)**. Molecular Functions, **(B)**. Cellular Components, and **(C)**. Biological Processse.

The GO database, established by the Association of Gene Ontology (GO) Consortium, encompasses three primary categories: Molecular Function (MF), Cellular Component (CC), and Biological Process (BP). MF analysis indicated that the target circadian genes enriched the functions of transcription coactivator activity, enzyme activator activity, RNA binding, and nucleic acid binding. CC analysis showed that the target circadian genes were mainly located in the chromatin, nucleolus, chromosome, lytic vacuole, and lysosome vacuole. BP analysis of the 338 target genes showed that these genes were mainly involved in response to xenobiotic stimulus, organonitrogen compound biosynthetic process, embryonic organ development, sensory organ development, cellular response to cytokine stimulus, response to cytokine, chemical homeostasis, animal organ morphogenesis, developmental growth, and response to chemical (**Figures 2A-C**).

### Pathway analysis

**Figure 3:**
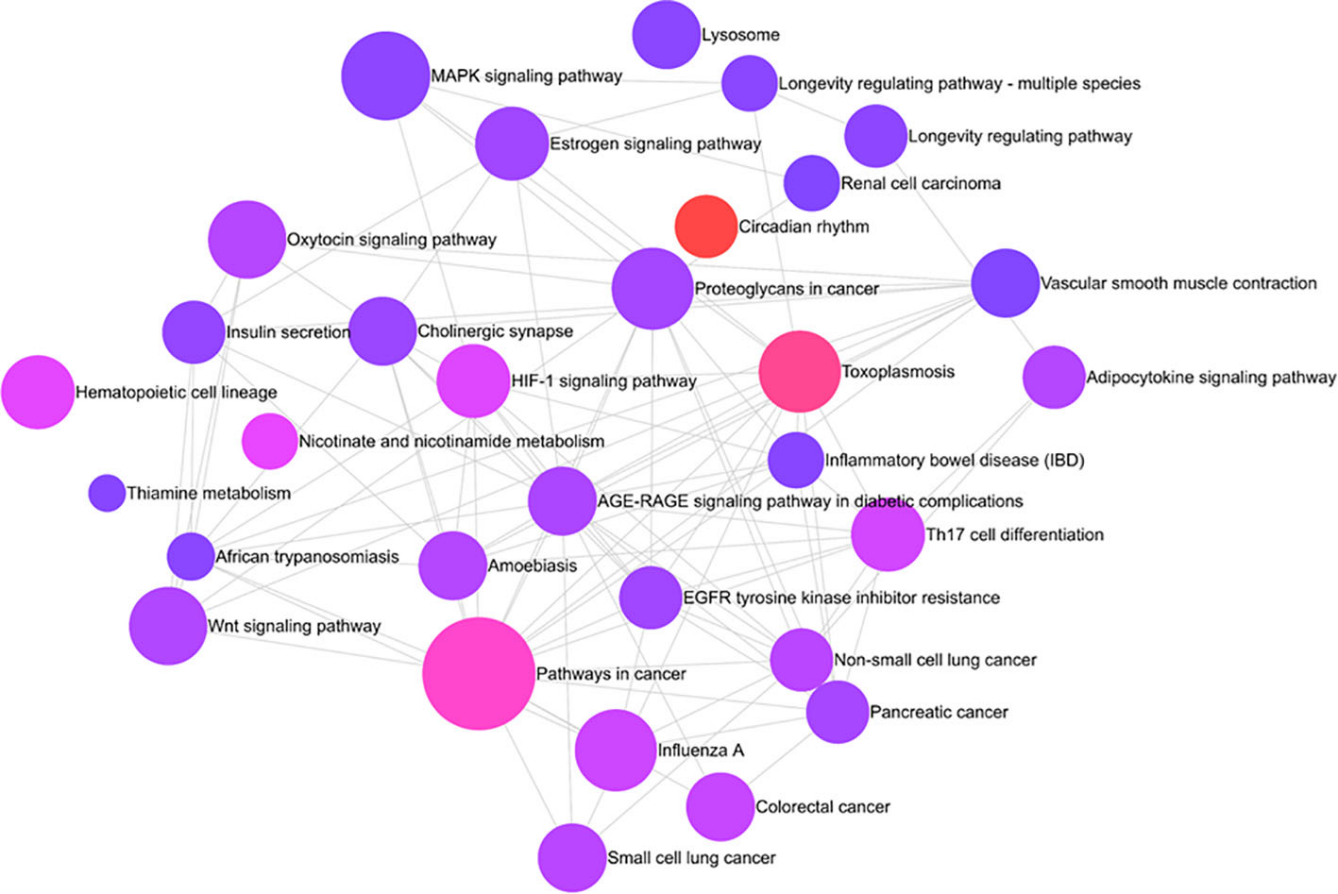
REACTOME chart of enriched pathways associated with HS: Interleukin 4/IL-13 signaling pathway, Interleukin-20 family signaling, SHC1 events in ERBB2 signaling, Platelet activation signaling and aggregation, Extra-nuclear estrogen signaling, and interferon-gamma signaling are a few among 10 pathways. The signaling by interleukin is the most enriched pathway with a p-value of less than 0.00005.

Pathway analysis of differentially methylated circadian genes linked to HS cases performed with REACTOME revealed enrichment of several prominent canonical cellular pathways, including MAPK signaling pathway, estrogen signaling pathway, oxytocin signaling pathway, WNT signaling pathway, as well as pathways associated with cancer (pancreatic and non-small cell lung cancer), longevity regulation, lysosomes, and insulin secretion (**Figure 3**).

### STRING network analysis of differentially methylated genes in HS patients

To decipher the potential interactions and associations between the 338 identified genes, which were differentially methylated in HS patients, we performed an in-depth STRING network analysis. The full STRING network was employed to map the interactions using multiple sources like text mining, experiments, databases, and co-expression, with a medium confidence threshold set at 0.400. Such a comprehensive approach was deemed necessary to gain an exhaustive understanding of the direct and indirect relationships between these genes.

The network resulting from the analysis was constituted by 338 nodes, corresponding to 338 genes from our dataset. Interestingly, the network presented a total of 568 edges, hinting at an intense degree of interaction among the selected genes. To place this in perspective, the average node degree stood at 3.37, suggesting that, on average, each gene interacted with around three other genes in this network. Furthermore, the average local clustering coefficient was found to be 0.348. This value indicates a moderate level of clustering between the genes, potentially hinting at functional modules or sub-networks within the larger interaction network. A particularly striking observation was the contrast between the expected and observed number of edges. While the expected number of interactions was 487, the observed interactions (568) significantly exceeded this prediction. The PPI enrichment p-value of 0.000176 further supported this observation, suggesting that the interactions within this network are biologically meaningful and not mere random associations.

**Figure 4:**
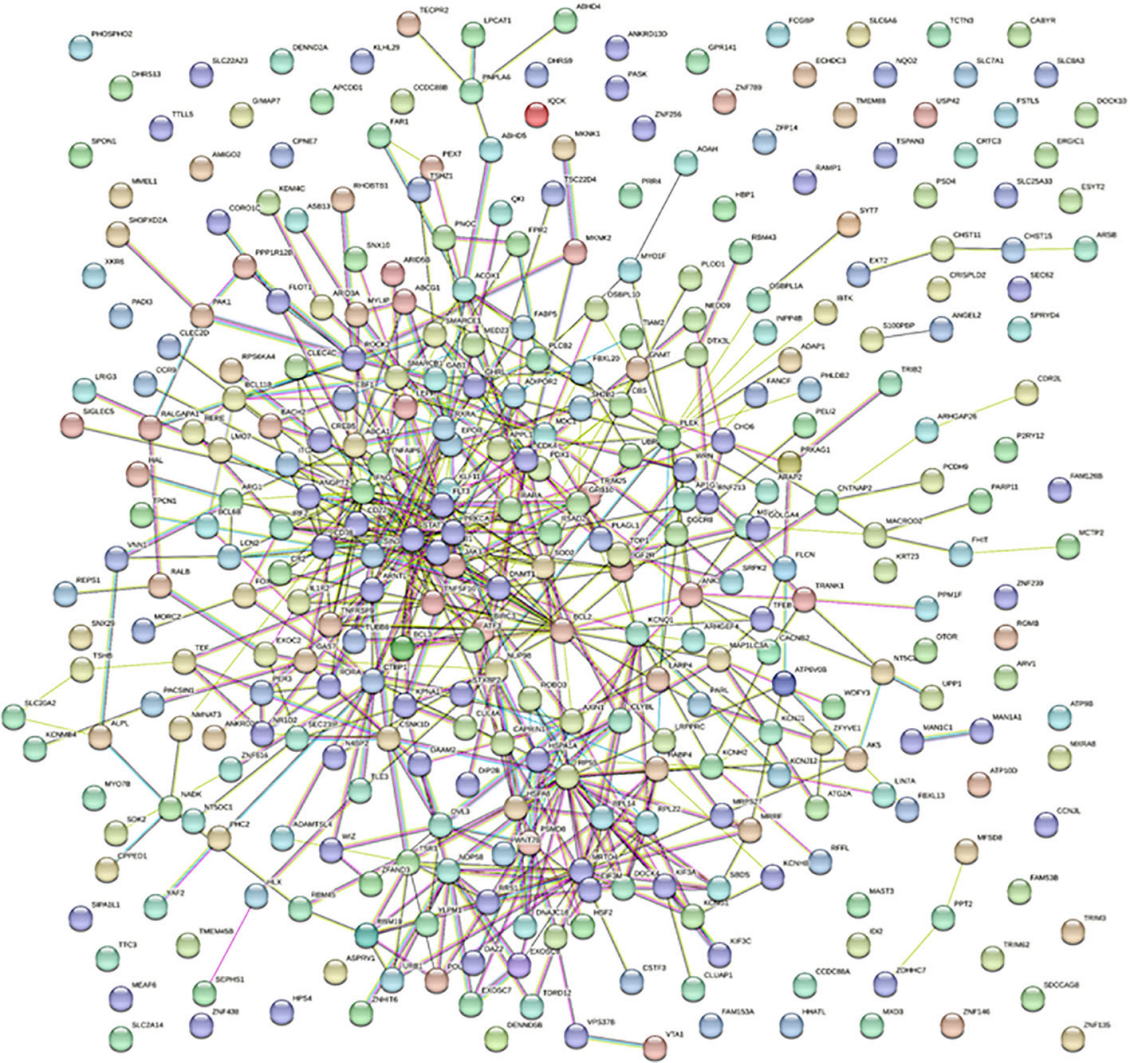
STRING protein-protein interaction networks for the differentially methylated circadian pathways-related genes in HS. The STRING network enables the assessment and integration of protein-protein interactions, both directly (physical) and indirectly (functional). Colored lines between proteins designate different types of interactions. Different colored lines represent different types of evidence used to predict associations. Red lines indicate fusion evidence; green lines, neighborhood evidence; blue lines, cooccurrence evidence; purple lines, experimental evidence; yellow lines, text mining evidence; light blue lines, database evidence; black lines, co-expression evidence.

Among the significant pathways, the “Circadian Rhythm” pathway (hsa04710) was of particular interest. The circadian pathway plays a pivotal role in regulating the body’s homeostasis, driving the daily patterns of behavioral changes and physiological processes. Out of the analyzed genes, 5 were associated with the “Circadian Rhythm” pathway. For context, the STRING database associates 30 genes with this pathway. The strength score of 0.99 reaffirms very strong confidence in this association, bolstering our initial hypothesis about the importance of circadian rhythm disruption in HS onset. Furthermore, an FDR of 0.0471 confirms the robustness of this association. The matching proteins from our dataset that were associated with this pathway are *RORA, PRKAG1, CSNK1D, ARNTL*, and *PER3*. Specifically, *RORA* had a node degree of 6, *PRKAG1* had 3, *CSNK1D* had 10, *ARNTL* had 9, and *PER3* had 5. These interactions, as suggested by their respective node degrees, might signify a potential coordinated response in HS’s development and progression. The observed dense interconnectivity might represent an adaptive biological response to disrupted circadian rhythms or, contrastingly, could be an exacerbating factor in this skin disease (**Figure 4**).

### Pathway analysis of differentially methylated circadian and ultradian genes

Exploiting RhythmicDB software with the primary dataset (GSE11923), 53, 237, and 3320 Ensembl Transcript IDs were retrieved for the 8-h, 12-h, and 24-h oscillating gene sets, respectively. We found differentially methylated CpGs within 338 circadian genes, comprising 271 hypomethylated and 67 hypermethylated genes in HS patients compared to controls, with a significance threshold adjusted for false discovery rate (FDR) at p-value < 0.05. Regarding ultradian genes, we found differentially methylated CpGs within 49 12h oscillating genes, with 39 hypomethylated and 10 hypermethylated genes. Interestingly, we found differentially methylated CpGs within 17 ultradian genes oscillating with 8-hour periodicity and all of them were hypomethylated, confirming the prevalence of hypomethylation of oscillating genes in HS patients.

Significantly differentially methylated 24-h oscillating genes enriched the following activated pathways: Xenobiotic Metabolism Signaling, Senescence Pathway, NRF2-mediated Oxidative Stress Response, Xenobiotic Metabolism General Signaling Pathway, Pulmonary Fibrosis Idiopathic Signaling Pathway, HIF1α Signaling, Xenobiotic Metabolism CAR Signaling Pathway, Neurotrophin/TRK Signaling, Autophagy. Among the enriched diseases and functions some were inhibited (Organismal Injury and Abnormalities, Organismal Survival, Developmental Disorder), and others were activated (Infectious Diseases, Organismal Injury and Abnormalities, Cellular Assembly and Organization, Cellular Function and Maintenance, Cellular Development, Cellular Growth and Proliferation, Cellular Movement, Cancer, Organismal Injury and Abnormalities, Lipid Metabolism, Small Molecule Biochemistry). The most representative networks were related to Gastrointestinal Disease, Hepatic System Disease, Liver Steatosis, Developmental Disorder, Hereditary Disorder, Metabolic Disease, Cancer, Organismal Injury and Abnormalities, Reproductive System Disease, Cell Death and Survival, Embryonic Development, RNA Post-Transcriptional Modification, Amino Acid Metabolism, Drug Metabolism, Small Molecule Biochemistry, Cellular Assembly and Organization, Hair and Skin Development and Function, Hereditary Disorder, Cellular Assembly and Organization, Cellular Function and Maintenance, Infectious Diseases (**Supplementary Table S1**). Among the selected core clock genes (*ARNTL, ARNTL2, CLOCK, CRY1, CRY2, DBP, HLF, NFIL3, NPAS2, NR1D1, NR1D2, PER1, PER2, PER3, RORA, RORC, TEF), ARNTL2, PER3* and *TEF* resulted hypomethylated, while *ARNTL, NR1D2* and *RORA* resulted hypermethylated (**Supplementary Table S2**).

Significantly differentially methylated 12-h cycling genes enriched the following pathways: Hypoxia Signaling in the Cardiovascular System, UDP-N-acetyl-D-glucosamine Biosynthesis, Unfolded protein response, Protein Ubiquitination Pathway, Glycogen Degradation, GDP-glucose Biosynthesis, p38 MAPK Signaling. The enriched diseases and functions were Cancer of the Head, Organismal Injury and Abnormalities, Cancer of the Large Intestine, Gastrointestinal Disease, Organismal Injury and Abnormalities, Cell Death and Survival, Hair and Skin Development and Function. The most representative networks were related to Cell Death and Survival, Molecular Transport, Protein Trafficking, Neurological Disease, Organ Morphology, Organismal Injury and Abnormalities, Developmental Disorder, Hereditary Disorder, Neurological Disease (**Supplementary Table S3**).

**Figure 5:**
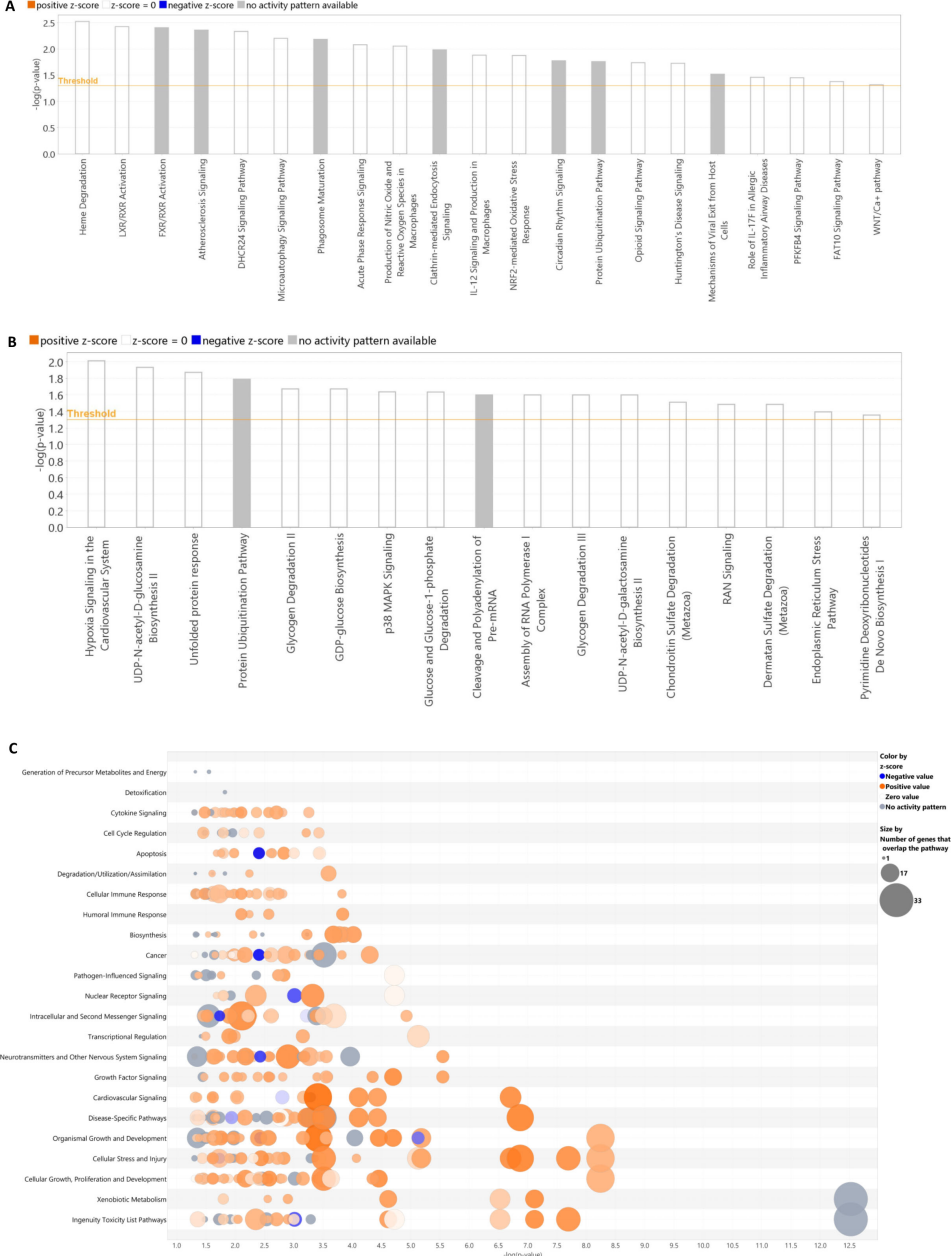
Pathway analysis for genes oscillating with circadian and ultradian periodicity resulting from IPA applied to the differentially methylated cycling genes. Oscillating transcripts with 8-h, 12-h, and 24-h periods are shown in **(A–C)**, respectively.

Significantly differentially methylated 8-h oscillating genes enriched the following pathways: Heme Degradation, LXR/RXR Activation, FXR/RXR Activation, micro autophagy and Phagosome Maturation Signaling Pathway, Acute Phase Response Signaling, Production of Nitric Oxide and Reactive Oxygen Species in Macrophages, Clathrin-mediated Endocytosis Signaling, IL-12 Signaling and Production in Macrophages, NRF2-mediated Oxidative Stress Response. The enriched diseases and functions were Lipid Metabolism, Molecular Transport, Small Molecule Biochemistry, Protein Synthesis, Free Radical Scavenging, Molecular Transport, Cell Death and Survival, Organismal Injury and Abnormalities, Connective Tissue Development, and Function, Embryonic Development, Organismal Development, Skeletal and Muscular System Development and Function, Tissue Development. The most representative networks were related to Lipid Metabolism, Molecular Transport, Small Molecule Biochemistry and Free Radical Scavenging, Nervous System Development and Function, Visual System Development and Function (**Supplementary Table S4**). A comprehensive report of IPA results is presented in **Figure 5** and **Supplementary Table S5**.

## Discussion

Our analysis of differential patterns of methylation of circadian and ultradian genes in peripheral blood specimens of HS patients compared to control subjects showed interesting results. First, most of the genes whose expression occurs cyclically were typically hypomethylated, which suggests a possible greater activation of the signaling pathways that they enrich, such as the MAPK signaling pathway and WNT signaling pathway, and of the cellular functions they control, such as response to xenobiotic stimulus, cellular response to cytokine stimulus, chemical homeostasis.

MAPK and WNT signaling pathways cooperate in maintaining tissue homeostasis and patterning in response to various extracellular signals and waving molecules, i.e. mitogenic stimuli such as growth factors, cytokines, and stress, to regulate cell proliferation, skin homeostasis, and epidermal stem cell dynamics and fate. On the other hand, MAPKs contribute to the regulation of WNT pathway activities and interactions between dysfunctional MAPK and WNT signal transduction systems might play a crucial role in the pathogenic mechanisms running skin diseases (48).

Considering the differentially methylated genes oscillating with 8-hour periodicity, only hypomethylation was found and this pattern impacted pathways such as degradation of heme, activation of cellular signaling dependent on *LXR/RXR* and *FXR/RXR* nuclear receptors, response to oxidative stress mediated by NRF2 and inflammation events related to acute phase response with production in macrophages of IL-12, the main mediator of the early phases of innate immune responses to intracellular microorganisms (49).

Regarding the cycling transcripts expressed with 12-hour periodicity with differential methylation pattern, they enriched relevant signaling pathways, such as unfolded protein response, glycogen degradation, GDP-glucose biosynthesis, and p38 MAPK signaling, which is crucial for the control of cytokines release by macrophages and neutrophils (50).

Among the transcripts oscillating with 24-hour periodicity, the core clock genes *ARNTL2, PER3*, and *TEF* resulted in hypomethylated, while *ARNTL, NR1D2*, and *RORA* resulted in hypermethylated. Differentially methylated 24-h oscillating genes enriched several activated pathways, such as the metabolism of xenobiotics, NRF2-mediated oxidative stress response, HIF1α signaling pathway, and autophagy. All these cellular functions and processes are tightly controlled by circadian pathways and the deregulation of the molecular clockwork impacts them in several physiopathological mechanisms of disease (51–53).

### Limitations

Despite these positive findings, our study acknowledges certain limitations. The complexity of epigenetic regulation and the multifactorial nature of the biological clock in controlling immune and inflammatory processes require deeper exploration. The derangement of circadian and ultradian pathways in HS, while significant, remains incompletely understood, necessitating further research to delineate specific mechanisms and their impact on disease progression. Additionally, the heterogeneity in patient populations and varying stages of HS may have influenced our findings, underscoring the need for larger, more diverse cohorts in future studies.

Moreover, the translational potential of these epigenetic insights into clinical interventions demands further validation. Functional studies are needed to establish causal relationships between the identified epigenetic markers and disease outcomes. Similarly, clinical trials will be essential to evaluate the efficacy of any potential therapeutic strategies that arise from these findings. Another limitation of our study is that, although 20-30% of HS cases involve patients with a family history, those individuals were not actively excluded; this occurred by coincidence rather than intentional exclusion. As a result, our cohort may not fully represent the broader HS population, including those with a genetic predisposition, potentially limiting the generalizability of our findings. Future research should account for familial history to better capture its role in HS.

### Conclusions

The changes in DNA methylation patterns found in the peripheral blood of HS patients could provide new insights into the molecular mechanisms underlying this chronic skin disease. Our bioinformatics analysis of significant differences in methylation patterns of transcripts oscillating with 24-h rhythmicity or with harmonics of circadian periodicity highlighted alteration of canonical signaling cascades, such as MAPK and WNT signaling pathways, as well as deregulated functional processes and activated pathways such as xenobiotic metabolism, oxidative stress, and hypoxia response, unfolded protein response, protein ubiquitination, acute phase response, protein synthesis, free radical scavenging, molecular transport, DNA damage response, cell death and survival, and firstly, hair and skin development and function.

Notwithstanding growing evidence of the pivotal role played by MAPK and WNT signaling pathways in the regulation of cell activities and processes, as well as tissue functions, and apart from their involvement in pathogenic mechanisms when deregulated, their interaction networks and potential roles in disease are still not fully appreciated, especially in the setting of skin diseases.

Exploration of methylome dysregulation in HS patients could contribute to enlightening the dysfunctional circuits and molecular networks that fuel HS onset and progression, but further investigation is necessary before being able to validate reliable biomarkers and molecular targets to identify innovative therapeutic strategies for HS patients.

## Data Availability

The original contributions presented in the study are included in the article/**Supplementary Material**, further inquiries can be directed to the corresponding author.

## Supplementary material

Supplementary Figure 1 Analysis of the Receiver operating characteristic (ROC) curve ≥0.95 for four circadian genes associated with HS. False Detection Rate (FDR) p-values for the methylation difference between HS subjects and controls were highly significant at each locus. AUC: Area Under the Receiver Operating Characteristics Curve; 95% CI: 95% Confidence Interval. Lower and upper confidence intervals are given in parentheses.

Supplementary Table 5 Results of Ingenuity Pathway Analysis performed on 24h, 12h, and 8h oscillating genes.
