# Supplementary figures and images for: DNA methylation patterns of circadian and ultradian genes are altered in the peripheral blood of patients with hidradenitis suppurativa
Source: Front Immunol. 2024 Nov 26;15:1475424. doi: 10.3389/fimmu.2024.1475424 (PMC11635105; doi:10.3389/fimmu.2024.1475424)

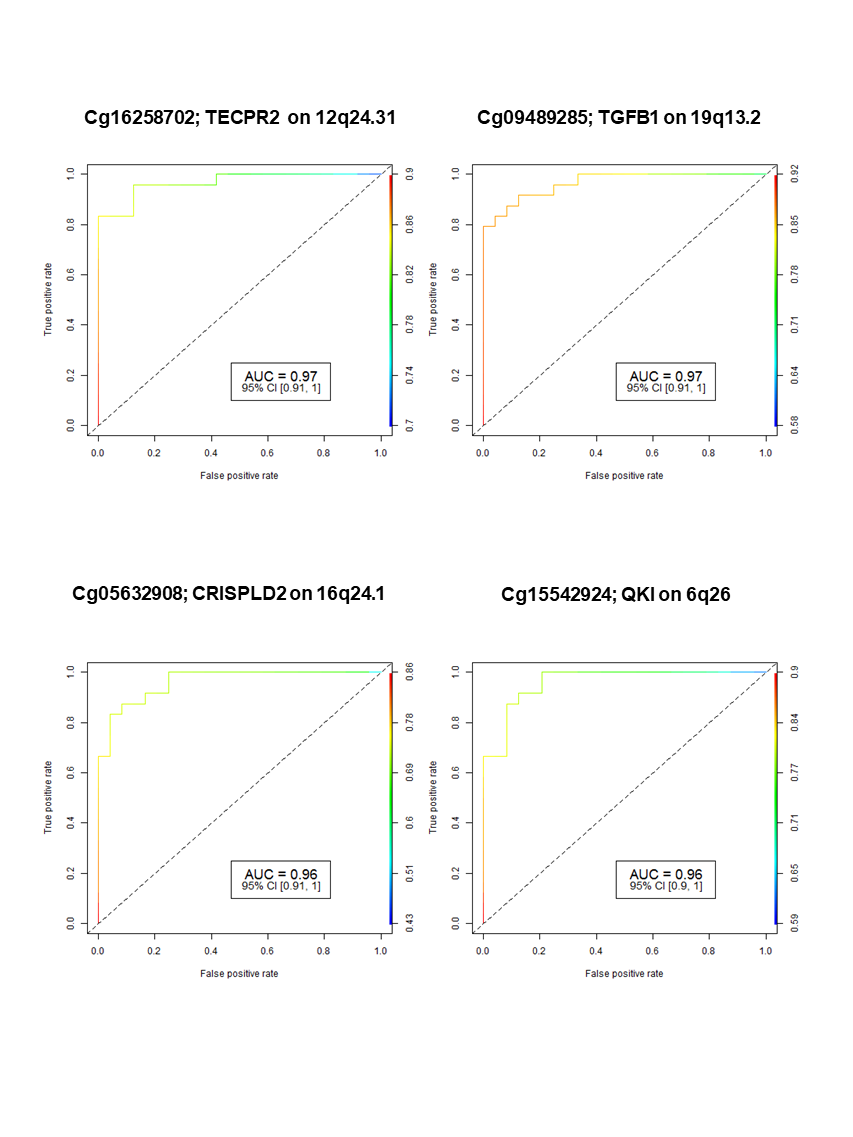

Supplement: Supplementary Figure 1 — Analysis of the Receiver operating characteristic (ROC) curve ≥0.95 for four circadian genes associated with HS. False Detection Rate (FDR) p-values for the methylation difference between HS subjects and controls were highly significant at each locus. AUC: Area Under the Receiver Operating Characteristics Curve; 95% CI: 95% Confidence Interval. Lower and upper confidence intervals are given in parentheses. [file Image1.tif]
